# High confidence ATFS-1 target genes for quantifying activation of the mitochondrial unfolded protein response

**DOI:** 10.17912/micropub.biology.000484

**Published:** 2021-10-19

**Authors:** Sonja K Soo, Jeremy M Van Raamsdonk

**Affiliations:** 1 Department of Neurology and Neurosurgery, McGill University, Montreal, Quebec, Canada; 2 Metabolic Disorders and Complications Program, and Brain Repair and Integrative Neuroscience Program, Research Institute of the McGill University Health Centre, Montreal, Quebec, Canada; 3 Division of Experimental Medicine, Department of Medicine, McGill University, Montreal, Quebec, Canada; 4 Department of Genetics, Harvard Medical School, Boston, MA, USA

## Abstract

The mitochondrial unfolded protein response (mitoUPR) is an evolutionarily conserved pathway that restores homeostasis to the mitochondria after various disturbances. This pathway has roles in both resistance to exogenous stressors and longevity. The mitoUPR is mediated by the transcription factor ATFS-1/ATF-5, which modulates the expression of genes involved in protein folding, metabolism and stress resistance. MitoUPR activation in *C. elegans* is most commonly evaluated through transcriptional reporter strains for the mitochondrial chaperones HSP-6 and HSP-60. In order to obtain a more comprehensive view of transcriptional changes resulting from activation of the mitoUPR, we compared gene expression changes from three different mitoUPR-activating interventions: mutation of *nuo-6, *RNA interference (RNAi) knockdown of *spg-7*,**and constitutive activation of ATFS-1. We specifically focused on gene expression changes that are dependent on ATFS-1. From this comparison, we identified 61 high confidence target genes that can be used to monitor mitoUPR activation. Notably, neither *hsp-6 *nor *hsp-60 *were significantly upregulated under all three mitoUPR activating conditions. We ranked the 61 genes according to the magnitude of upregulation and identify multiple genes that may serve as robust readouts of mitoUPR activation.

**Figure 1. Levels of gene expression under conditions of mitoUPR activation:**
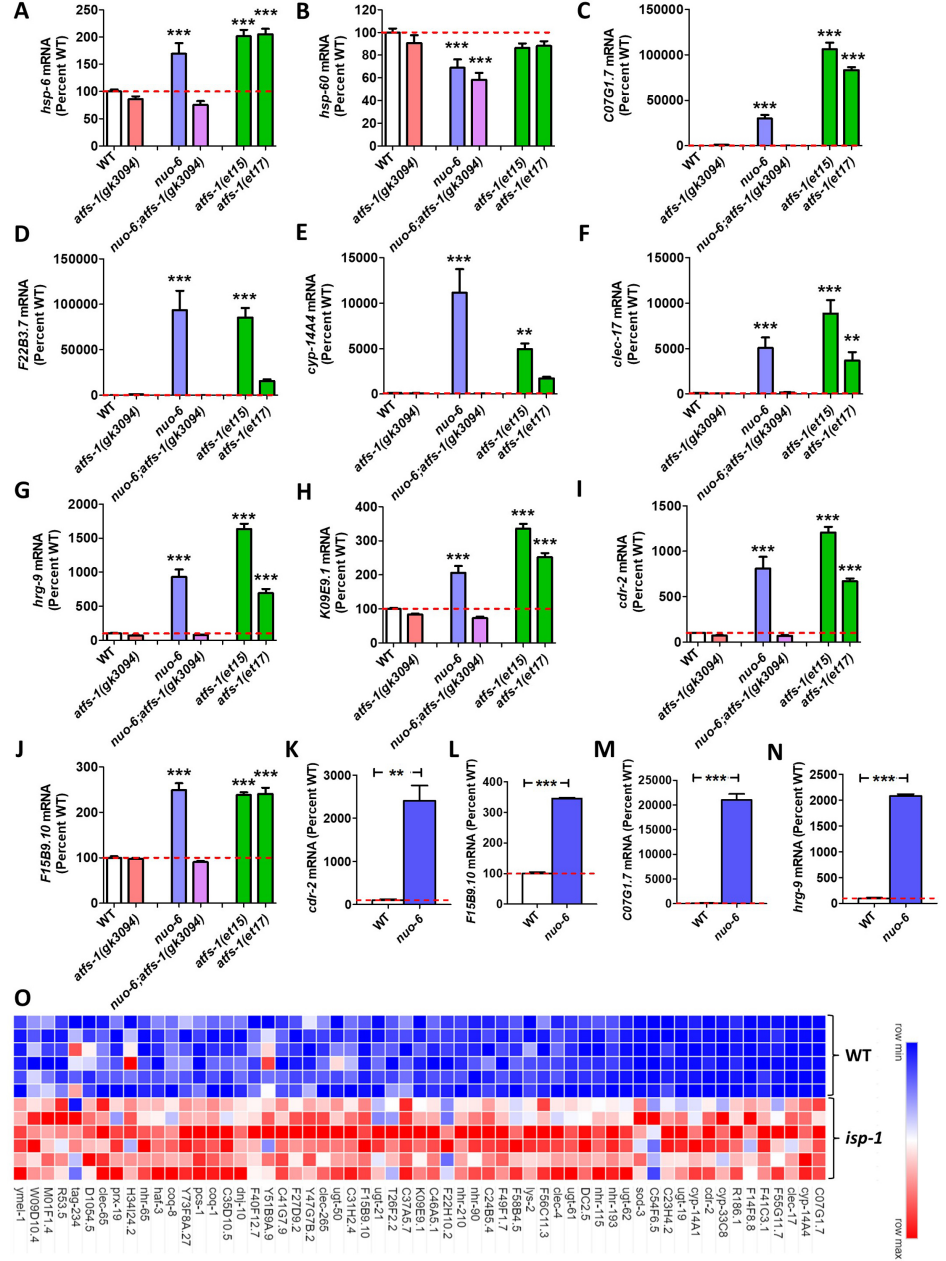
To compare the efficacy of using different target genes to monitor activation of the mitoUPR, we examined the expression of each gene under conditions of ATFS-1 activation. To activate ATFS-1, we used a *nuo-6* mutation (blue bars) or the constitutively active *atfs-1(et15)* and *atfs-1(et17)* mutations (green bars)*.* ATFS-1 activation was prevented using an *atfs-1* deletion mutation *gk3094* in wild-type and *nuo-6* worms (red bars and purple bars, respectively). Expression is shown as a percentage of wild type worms (white bars). **A.** There is a significant increase in *hsp-6* mRNA under conditions of ATFS-1 activation that is prevented by disruption of *atfs-1.* **B.** The levels of *hsp-60* are not increased in *nuo-6* mutants or either constitutively active *atfs-1* mutants. **C-F.**The mitoUPR target genes *C01G1.7, F22B3.7, cyp-14A4* and *clec-17* exhibit a marked increased in expression under conditions of ATFS-1 activation with a magnitude much greater than *hsp-6.* **G-J.** The mitoUPR target genes *hrg-9, K09E9.1, cdr-2* and *F15B9.10* show a large increase in expression relative to their standard deviation under conditions of ATFS-1 activation. **K-N.** The expression of mitoUPR target genes *cdr-2, F15B9.10, C07G1.7* and *hrg-9* show increased expression in *nuo-6* worms by quantitative RT-PCR. **O.** Heat map comparing the expression of identified mitoUPR target genes between wild-type and long-lived mitochondrial mutant *isp-1* worms. All but 4 of the 61 genes are significantly upregulated in *isp-1* mutants. Expression levels in panels A-J were determined from our previously published RNA sequencing data (Wu *et al.*, 2018). Expression represents counts per million from six biological replicates that has been normalized to wild-type. Heat map was generated using our previously published RNA sequencing data (Senchuk *et al.*, 2018). Error bars indicates SEM. **p<0.01, ***p<0.001. Statistical significance was assessed using a one-way ANOVA with Dunnett’s multiple comparison test, except in panels K-N where a student’s t-test was used.

## Description

The mitochondrial unfolded protein response (mitoUPR) is a stress response pathway that promotes cell survival and restores mitochondrial function when mitochondrial health is compromised (Haynes *et al.* 2013; Jovaisaite *et al.* 2014; Shpilka and Haynes 2018). While a mitoUPR was first reported in mammalian cells (Zhao *et al.* 2002), the initial work on the mitoUPR in *C. elegans* was performed by Yoneda *et al.* who found that treatment with ethidium bromide, which affects the replication and expression of mitochondrial DNA, increased the expression of the mitochondrial chaperone gene *hsp-6* (Yoneda *et al.* 2004). Based on this observation, they generated *hsp-6p::gfp* and *hsp-60p::gfp* reporter strains to further study the mitoUPR. They found that either RNA interference (RNAi) targeting *spg-7,* the worm homolog of paraplegin, or RNAi targeting other genes encoding mitochondrial proteins resulted in activation of the *hsp-6p::gfp* and *hsp-60p::gfp* reporter strains (Yoneda *et al.* 2004).

The *hsp-6p::gfp* and *hsp-60p::gfp* reporter strains were used to identify other components of the mitoUPR including the transcriptional regulator UBL-5, the transcription factor DVE-1, the protease ClpP, the protein import channel HAF-1, and the transcription factor ATFS-1 (Benedetti *et al.* 2006; Haynes *et al.* 2007; Haynes *et al.* 2010; Nargund *et al.* 2012). Under normal conditions, the mitoUPR transcription factor, ATFS-1, is imported into the mitochondria through the HAF-1 import channel and degraded by the protease ClpP. However, when mitochondria are impaired, ATFS-1 import into the mitochondria is blocked. Instead, the nuclear localization signal (NLS) of ATFS-1 drives ATFS-1 into the nucleus. In the nucleus, ATFS-1 acts with DVE-1 and UBL-5 to upregulate genes that restore mitochondrial homeostasis, including genes involved in mitochondrial protein folding and metabolism. In addition to responding to disruptions in mitochondrial function and integrity, the mitoUPR also plays important roles in resistance to stress (Pellegrino *et al.* 2014; Campos *et al.* 2021; Soo *et al.* 2021) and longevity (Durieux *et al.* 2011; Houtkooper *et al.* 2013; Bennett *et al.* 2014; Wu *et al.* 2018).

Previous studies from our laboratory and others have used RNA sequencing (RNA-seq) or microarrays to examine gene expression under conditions that induce ATFS-1 activation. Nargund *et al.* identified 366 genes that are upregulated by *spg-7* RNAi in an ATFS-1-dependent manner (Nargund *et al.* 2012). We previously identified 1704 genes that are upregulated in *nuo-6* mutants in an ATFS-1-dependent manner (Senchuk *et al.* 2018; Wu *et al.* 2018) and 529 genes that are upregulated in both *atfs-1(et15)* and *atfs-1(et17)* constitutively active *atfs-1* mutants (Rauthan *et al.* 2013; Wu *et al.* 2018).

In order to establish a list of mitoUPR target genes that are consistently upregulated across all three ATFS-1-activating conditions, we examined the overlap between these datasets. We identified a total of 61 genes that are upregulated in *nuo-6* worms in an ATFS-1-dependent manner, upregulated by *spg-7* RNAi in an ATFS-1-dependent manner, and upregulated in both *atfs-1(et15)* and *atfs-1(et17)* constitutively active mutants (**Extended data, Column A,B**). Surprisingly, neither *hsp-6* nor *hsp-60* were among the 61 genes on this list. In fact, *hsp-60* was not identified in any of the three datasets. It is uncertain why *spg-7* RNAi increases fluorescence in the *hsp-60p::GFP* reporter strain (Yoneda *et al.* 2004) but *hsp-60* was not identified as one of the genes upregulated by *spg-7* RNAi (Nargund *et al.* 2012). One possibility may be that *hsp-60* expression is primarily upregulated during development in response to *spg-7* RNAi and other mitochondrial insults. Then the half-life of the HSP-60::GFP protein might allow the increase in HSP-60::GFP to still be observed at adulthood even after the *hsp-60* mRNA has returned to baseline.

In order to rank the different mitoUPR target genes, each gene was scored by adding together their expression levels in *nuo-6, atfs-1(et15)* and *atfs-1(et17)* mutants and subtracting their expression level in *nuo-6;atfs-1* mutants. By this metric, 42 of the 61 mitoUPR target genes exhibited a higher score than *hsp-6* (**Extended data, Column J**)*.* The top genes of *C07G1.7*, *F22B3.7, cyp-14A4,* and *clec-17* had scores ranging from 17084-21882, compared to a score of 350 for *hsp-6.* The expression of these genes compared to *hsp-6* and *hsp-60* is shown in **Fig 1A-F***.*

In order to control for variability, we divided this score by the average standard deviation from WT, *atfs-1(gk3094), nuo-6, nuo-6;atfs-1(gk3094), afts-1(et15)* and *atfs-1(et17*). By this metric, 27 of the 61 mitoUPR target genes showed a higher score than *hsp-6* (**Extended data, Column K**)*.* The top genes of *C07G1.7*, *hrg-9, K09E9.1, cdr-2* and *F15B9.10* had variability-corrected scores of 26-38 compared to 15 for *hsp-6.* The expression of these genes is shown in **Fig 1G-J***.*

To determine if these mitoUPR target genes are being activated directly by binding of ATFS-1, we examined the data from a previous chromatin immunoprecipitation sequencing (ChIP-seq) experiment that identified genes bound by ATFS-1 after treatment with *spg-7* RNAi (Nargund *et al.* 2015). We found that 22 of the 61 mitoUPR target genes exhibited binding of ATFS-1 after *spg-7* RNAi (**Extended data, Column L**). This suggests that the expression of these genes is directly modulated by ATFS-1, while the expression of the other mitoUPR target genes may be regulated indirectly, or perhaps bound by ATFS-1 under different conditions than were utilized in the ChIP-seq study.

Our results suggest that the genes identified here may be better for monitoring the activation of the mitoUPR by quantitative RT-PCR or RNA-seq than *hsp-6* and *hsp-60.* To that end, we have designed and validated primers for quantitative RT-PCR to measure the levels of two of the highest-ranked direct targets, *cdr-2* and *F15B9.10,* and two of the highest ranked indirect targets, *C07G1.7* and *hrg-9.* We confirmed that all four sets of primers can efficiently measure activation of the mitoUPR in *nuo-6* worms compared to wild-type worms (**Fig 1K-N**).

Finally, to validate this list of 61 genes for monitoring the activation of the mitoUPR, we examined the long-lived mitochondrial mutant *isp-1,* which we have previously shown to have increased activation of a *hsp-6p:::gfp* reporter strain and significantly overlapping gene expression changes with constitutively active *atfs-1* mutants (Wu *et al.* 2018). 57 of the 61 genes were found to be significantly upregulated in *isp-1* mutants (**Extended data,** **Fig 1O**).

Overall, this work has identified a gene expression signature that can be used to monitor the activation of the mitoUPR in RNA-seq, microarray or qPCR studies. These genes can be used to complement the use of *hsp-6p::gfp* and *hsp-60p::gfp* reporter strains, and may provide a more robust measure of mitoUPR activation in studies measuring mRNA.****

## Methods

**Strains**. Worms were grown on NGM plates seeded with OP50 bacteria at 20°C. We previously performed RNA sequencing on wild-type(N2), *atfs-1(gk3094)*, *nuo-6(qm200)*, *nuo-6(qm200)*;*atfs-1(gk3094)*, *atfs-1(et15)*, *atfs-1(et17)* and *isp-1(qm150)* worms (Senchuk et al. 2018; Wu et al. 2018). This RNA sequencing data was re-analyzed to generate Figure 1, panels A-J and Figure 1, panel O. The quantitative RT-PCR results in Figure 1, panels K-N were generated for this publication using wild- type and *nuo-6(qm200)* worms

**Quantitative RT-PCR.** Worms from an overnight limited lay were allowed to grow to the prefertile young adult stage. Worms were washed three times with M9 buffer and frozen in Trizol. RNA was isolated by phenol-chloroform extraction using Trizol reagent as previously described (Machiela *et al.* 2016). RNA was converted to cDNA using a High Capacity cDNA Reverse Transcription Kit (Life Technologies) according to the manufacturer’s protocol. qPCR was performed in a Viia 7 RT-PCR machine from Applied Biosystems real-time thermal cycler using PowerUp SYBR Green Master Mix kit (Applied Biosystems). RNA was collected from three biological replicates and normalized to the levels of *act-3.* Primer sequences:

*cdr-2* (L: CGAGCCTCATTTGGAAAGAA, R: GCATCTGCCGCTGTAACTTT)

*F15B9.10* (L: CCGGACAGTTTCAAGAATGC, R: CACTGAGGATCCAATGTCCA)

*hrg-9* (L: TGGAATATTGAGTGGCGTTG, R: CCTCCTCTACTTGGTGCATGT)

*C07G1.7* (L: GCTGAAGAAGCTTCAACCGTAG, R: TCTCGTGTCAATTCCGGTCT)

**Analysis of gene expression data.** Lists of differentially expressed genes were obtained from (Nargund *et al.* 2012;Wu *et al.* 2018). The raw data from these experiments is available on NCBI GEO (https://www.ncbi.nlm.nih.gov/geo/query/acc.cgi?acc=GSE38196, https://www.ncbi.nlm.nih.gov/geo/query/acc.cgi?acc=GSE110984). We used BioVenn (https://www.biovenn.nl/index.php) to generate lists of overlapping genes between gene sets. Genes upregulated in *nuo-6* worms in an ATFS-1-dependent manner are genes that are upregulated in *nuo-6* mutants but not *nuo-6;atfs-1* mutants. To rank mitoUPR target genes, expression of each gene was normalized to wild-type. The score for each gene was determined by summing the normalized expression in *nuo-6, atfs-1(et15)* and *atfs-1(et17)* mutants and subtracting three times the expression in *nuo-6;atfs-1* mutants. To generate a variability-corrected score, this score was divided by the average standard deviation for each of the strains. Binding of ATFS-1 was determined from a previous chromatin immunoprecipitation (ChIP) study (Nargund *et al.* 2015). Data from this study is available on NCBI GEO: (https://www.ncbi.nlm.nih.gov/geo/query/acc.cgi?acc=GSE63803).

## Reagents

**Table:** 

**Strain**	**Genotype**	**Source**
N2	wild-type	Wild isolate from Bristol
MQ1333	*nuo-6(qm200)*	Yang, Hekimi. 2010.
VC3201	*atfs-1(gk3094)*	*C. elegans* Reverse Genetics Core Facility at the University of British Columbia
QC115	*atfs-1(et15)*	Rauthan et al. 2013.
QC117	*atfs-1(et17)*	Rauthan et al. 2013.
MQ887	*isp-1(qm150)*	Feng et al. 2001.
JVR477	*nuo-6(qm200); atfs-1(gk3094)*	Wu et al. 2018.
